# Securing independence in global health oversight—the OPEN framework

**DOI:** 10.1093/haschl/qxaf231

**Published:** 2025-11-27

**Authors:** Nina Schwalbe, Elliot Hannon, Susanna Lehtimaki, Brian Wahl

**Affiliations:** Center for National and Global Health Policy and Politics, Georgetown University, Washington DC 20001, United States; Spark Street Advisors, New York, NY 10013, United States; Spark Street Advisors, New York, NY 10013, United States; Spark Street Advisors, New York, NY 10013, United States; Spark Street Advisors, New York, NY 10013, United States; Department of Epidemiology of Microbial Diseases, Yale School of Public Health, New Haven, CT 06510, United States

**Keywords:** public health, global health, health policy, global health governance, oversight mechanisms, functional independence

## Abstract

The credibility of global health oversight mechanisms relies on their perceived independence. What truly constitutes “independent,” however, remains ill-defined. Using a mixed-methods approach that includes a literature review and 54 key informant interviews, this paper outlines 4 pillars of independence: operational, political, economic, and knowledge/technical. It then proposes a practical tool for evaluating their application—the “OPEN Framework.” We tested this framework by reviewing it against 3 so-called independent monitoring bodies: the Global Polio Eradication Initiative's Independent Monitoring Board, the Global Preparedness Monitoring Board, an independent monitoring and accountability body to ensure preparedness for global health crises, and the Independent Accountability Panel for Maternal, Newborn, and Child Health. Our findings reveal that, despite intentions of independence, pragmatic constraints and dependencies often compromise autonomy. The paper argues for a shift from rhetorical to operational independence by applying this framework, identifying conflicts of interest, and actively managing them. The OPEN Framework offers a replicable methodology for evaluating, comparing, and enhancing the independence of oversight bodies, thereby fostering stronger accountability and trust in global health governance.

Key Points

- Independence in global health oversight is often claimed but rarely defined or achieved. Oversight bodies face systemic constraints, including conflicts of interest and funding dependencies that compromise their autonomy.
- Our research and analysis identify 4 critical dimensions—organizational/operational, political, economic/financial, and knowledge/technical—that are essential for functional independence (OPEN). We propose the OPEN Framework to set safeguards and guidelines that can standardize practice and increase the credibility, transparency, and accountability of these bodies.
- The OPEN Framework offers a replicable tool to evaluate and strengthen independence in global health accountability mechanisms, moving beyond rhetoric toward operational independence.

## Introduction

The term “independence” is invoked frequently in public health discourse and beyond as an essential credential of trustworthiness. When an “independent board” commissions an “independent agency” to conduct an “independent review,” the findings are presumed to be objective and therefore more credible. Political scientists have studied the importance of perceived independence in international institutions, while noting that the precise definition remains unclear in many instances.^1^ Their work suggests that true independence involves nuanced considerations of autonomy, neutrality, and delegated authority—elements rarely addressed in common usage. This conceptual imprecision has gradually muddled the term's meaning and diminished its impact, prompting a fundamental question: what does independence genuinely signify in today's increasingly interconnected world?

Conceptually, independence refers to freedom from external influence, control, and pressure. But in practice, those bright theoretical lines get blurrier. In an era of accelerated globalization—characterized by intricate political networks, integrated financial systems, and densely woven social connections—one would logically expect genuine independence to become increasingly scarce, not more prevalent. Researchers of global governance have documented how the intensification of transnational ties creates structural constraints that complicate institutional autonomy.^2^

In global public health, there are overlapping spheres of power that often require different forms and expressions of independence. In global public health governance, the concept of independence exists within complex power dynamics and institutional frameworks. Oversight mechanisms are frequently established with the explicit mandate for establishing independence. However, there are often pragmatic constraints and challenges with regard to the application of these oversight mechanisms. The most fundamental of which is that independent assessments are often viewed as introducing unpredictable variables into high-stakes decision-making processes. For international bodies, these practicalities force a decision: embrace the unpredictability of genuine independence—with its potential to challenge established interests—or maintain more controlled oversight processes that preserve institutional stability but compromise the independence they purport to value.

This dichotomy manifests concretely in the foreign aid industry—a $135 billion competitive marketplace where UN agencies, development banks, and global foundations publicly champion independent monitoring while simultaneously navigating reputational risks. Their public statements emphasize independence as essential for integrity and credibility, yet operational realities reveal a reluctance to implement evaluation mechanisms that might expose shortcomings or suggest alternative approaches.^3^

This dynamic has fostered what some scholars have termed the “success cartel”—a tacit agreement among stakeholders to avoid political, financial, and reputational blowback that organizations or governments face when they fail to deliver on ambitious targets.^3^ The existential need for continued funding creates systemic pressures that compromise ostensibly “independent” oversight mechanisms. What emerges is a functional contradiction: accountability initiatives are labeled as independent while operating within power structures that fundamentally constrain their autonomy through financial dependencies and institutional relationships.

In this paper, we operationalize the concept of independence in global health oversight. Rather than accepting superficial “independent” labels, we propose that the global health community would benefit from acknowledging conflicts, understanding influence mechanisms, and managing them transparently. While numerous reports have highlighted threats to independence from donors and implementing partners,^4,5^ effective solutions remain elusive. We hope this pragmatic approach to operationalizing independence will enhance transparency, foster trust, and ultimately improve public health outcomes.

## Methods and approach

We followed a 3-phase mixed-methods approach with the objective of providing public health bodies a tool to better assess functional independence, applying that tool to current monitoring bodies to show the difference between theoretical and functional independence, and providing recommendations on better avenues to ensure and maintain independence.

First, we developed a framework for assessing independence based on evidence from oversight and accountability mechanisms across diverse international agreements.^6^ We assessed design principles for mechanisms that monitor adherence to international agreements, conventions, statutes, and regulations.^7,8^ This analysis was supplemented with primary data from 54 key informant interviews conducted between April 2021 and October 2023, including practitioners, academics, and policymakers with experience in global governance oversight. Through iterative, inductive thematic analysis of these data sources, we identified 4 critical dimensions of independence: organizational/operational, political, economic/financial, and knowledge/technical.

Second, we systematically applied this framework to evaluate global health initiatives that self-identify as independent. For each initiative, we examined governance structures, funding mechanisms, reporting relationships, decision-making processes, and operational autonomy. We assessed how each initiative's design either enables or constrains independence across the 4 dimensions of our framework. This evaluation included document analysis of terms of reference, governance charters, financial reports, and public statements.

Third, we identified independence deficits within these initiatives and developed tailored recommendations to strengthen functional independence. These recommendations acknowledge the practical realities of global health governance while providing actionable pathways to enhance transparency, mitigate conflicts of interest, and strengthen oversight effectiveness.

For the assessment, we reviewed publicly available materials, primarily terms of reference and official websites of the entities. Each entity was assessed across 4 predefined areas of independence using a structured tool developed for this purpose. Two reviewers independently conducted assessments based on a 3-point scale measuring independence: (1 = Yes, 3 = Partially/not sure, 5 = No). Any discrepancies in scoring were resolved through discussion on a consensus basis. Limitations include the potential influence of reviewer bias, which we sought to mitigate by using 2 independent reviews.

## Defining independence

While independence is definitionally the freedom from external influence, control, and pressure, what is clear from its application is that independence is a moving target that can change depending on the context. Independence is therefore a condition that must be established, actively secured, and maintained.

Once independence has been established, it suggests the ability to operate without being subject to political, organizational, and corporate authority.^9^ It also comes with the expectation of reasonable access to any information necessary and the autonomy to carry out inquiries to complete the given task. Independence enables impartiality through the ability to avoid conflicts of interest and external pressures that could compromise objectivity.

Independent oversight in public health and governing other international agreements typically involves multiple individuals forming a committee to carry out its assigned oversight task. How this committee is structured, selected, funded, and operates, all have important implications for its ability to assert—and maintain—its functional independence. As a precursor to a committee's independence, individual committee members must have the ability, expertise, and competence to conduct themselves independently. Maintaining members' independent disposition requires a package of terms and conditions to manage individual bias.

## Results

### Four dimensions of independence

Our analysis identified 4 distinct dimensions of independence that collectively determine the functional autonomy of oversight mechanisms in global health: Organizational/Operational, Political, Economic/Financial, and kNowledge/Technical independence. These pillars make up our OPEN Framework for evaluating functional independence. Each dimension of the framework represents an important pillar that must be sufficiently established and maintained for a monitoring body to provide genuinely independent oversight. While not always equally weighted, each pillar is necessary, but not sufficient, for independence.

#### Organizational and operational independence (O)

Organizational and operational independence is the committee's capacity to exert control over its operations,^10^ including sole discretion over its own financial and human resources, such as hiring of staff, travel, meetings, and administrative support, to maintain autonomy over its work.^11^ The committee's members carry out their duties, including producing and disseminating reports, without interference from organizational policies and hierarchies of the hosting/supporting organization(s) or external stakeholders.^10^ Organizational and operational independence can be impeded through the hosting arrangements of a given monitoring committee, where it is wholly or partially dependent on a single organization and its secretariat for its staffing and support. Operational independence can also be diminished when the committee's findings are expressed solely through the prism of this hosting/supporting body, giving the host the ability to overrule or moderate any unpopular findings.^10^ The committee's findings do not necessarily need to be reported publicly to be considered independent; they can, for instance, inform internal assessments that, in some instances, can have more impact.

#### Political independence (P)

Political independence is expressed through the committee's ability to carry out its work as it sees fit and deliver findings that are at odds with the interests of powerful players, such as countries, organizations, and other stakeholders (particularly foundations),^10^ without potentially threatening the other pillars of its independence. Creating the conditions for political independence includes clearly defined criteria for committee member appointment and dismissal and, where necessary, diplomatic immunity for carrying out official duties.^11^ The committee's decisions are made solely by its members, who participate in their individual capacities, not as representatives of their countries or organizations. Political independence is often manifested as freedom from individual interests and allegiances of committee members themselves, as well as power/influence exerted on the body as a whole to reach a specific conclusion.^10^

#### Economic and financial independence (E)

Financial independence is predicated on the secure and sufficient financing for a committee to carry out its mandate, which is not contingent on any specific outcome of its work. The funding must not be subject to preconditions through which countries and other donors can impose their individual priorities or political considerations.^12,13^ To help ensure this, funding should be derived from assessed or non-earmarked (either by states or private donors) funding, and the committee itself should be firewalled from engagement with donors.^14,15^ The threat of losing funding is the most pervasive form of influence in public health, capable of creeping into the monitoring and accountability work of seemingly independent bodies.

#### Knowledge and technical independence (N)

Technical independence constitutes the capacity of a monitoring committee to carry out its oversight function effectively. This means the committee is sufficiently configured and empowered^16^ to collect necessary information as needed and, where relevant, consult external sources to support or verify information.^10,11^ The mode of data collection can be passive, where the committee issues a public call for input or evidence, or active, where it has resources, such as access to experts, to generate new evidence.^17^ In some specific cases, this can include the authority to conduct announced or unannounced on-site visits to verify information.^16^ Technical independence is predicated on committee members possessing solid/sufficient technical qualifications and being transparent about conflicts of interest.^18^ In an instance where a committee is comprised in such a way that it does not have access to the required information to carry out its oversight mandate and does not have the ability to acquire that information, it will not have sufficient technical independence to render impartial, unbiased, independent assessments.

### Piloting the framework

We used our OPEN independence framework to evaluate 3 different monitoring mechanisms in global public health that purport to be independent: (1) the Global Polio Eradication Initiative's Independent Monitoring Board (IMB);^19^ (2) the Global Preparedness Monitoring Board (GPMB);^20^ and (3) the Independent Accountability Panel (IAP) for Maternal, Newborn and Child Health.^21^

The IMB, established in 2010 at the request of the WHO Executive Board and the World Health Assembly, oversees the WHO-led Global Polio Eradication Initiative (GPEI) and its work on the detection and interruption of poliovirus. It is comprised of global experts (currently 4 individuals) who serve in their personal capacity.^22,23^ The IMB monitors polio-endemic and outbreak-affected countries to assess the quality, implementation, and impact of their polio eradication plans, conducts related program performance and progress assessment, and advises countries, implementing partners, like UNICEF and the Centers for Disease Control and Prevention (CDC), as well as donor agencies, including the Gates Foundation, on how to improve performance. The IMB holds meetings typically once or twice a year when it reviews data derived from the GPEI, polio-affected countries, implementing partners, and donor agencies, and questions country and program leaders. It can also hold private meetings to evaluate progress toward key milestones.^1,5,6^ The IMB publishes their report and recommendations within 3 weeks of the meeting. There is no interference by the GPEI, other stakeholders, or member states. The report includes country-specific findings as well as broader aspects of the response that may change from year to year, such as regional and global responses, gender dimensions, and scientific advances.^24^ The work of the IMB is intended to contribute to global programming and has served to convince the World Health Assembly to declare polio eradication a programmatic emergency in 2012. It also considered to have enhanced country efforts by highlighting the poor performance of some national programs.^25^

In contrast to the IMB, the GPMB has a broad monitoring mandate to oversee collective and systemic preparedness to respond to global health crises. Established in 2018 after the Ebola outbreak by the WHO and World Bank, the GPMB reports on global progress on preparedness and response capacities for disease outbreaks and health emergencies to the general public. The GPMB has 15 members composed of political leaders, agency principals, and experts, including those from academia.^26^ The GMPB publishes annual reports focused on the previous year's successes and challenges in preparedness, including progress, if any, against its own monitoring framework, which consists of 90 indicators that track pandemic risks, capacities, and impacts at global, regional, and national levels.^27^ The report is intended to be used for advocacy with donors, member states, and other stakeholders to increase pandemic preparedness and response capacity, including, for example, at high-level political platforms such as the G20. The reports have been criticized for focusing on evidence that support WHO priorities and talking points and lacking concrete policy options to guide national and public health decision-making.^28^

The third example we explore in this analysis is Independent Accountability Panel for Women's Children's and Adolescent health (IAP) established by the UN Secretary General (UNSG) for the period 2016-2021 to oversee global and national efforts to fulfil the Sustainable Development Goals agenda under the Global Strategy for Women's, Children's, and Adolescents' Health.^29^ The IAP was hosted by the WHO-hosted Partnership for Maternal, Newborn, and Child Health (PMNCH), and it comprised 10 expert members, serving in their personal capacity and appointed by the UNSG.^30^ Its purpose was to identify and advocate actions to achieve the Strategy's goals. Using a range of sources, including from UN agencies, academia, civil society, and independent monitoring groups, such as national human rights institutions, the IAP monitored accountability to health and human rights. It then produced recommendations aimed at the international community.^30^ The findings were summarized in an annual report submitted to the High-Level Steering Group of the Strategy, chaired by the UNSG, and disseminated to stakeholders, including development partners, civil society, academia, donors, and the private sector. According to an external evaluation in 2019, the reports did not lead to a significant impact as the findings were disseminated through relatively small events, and there was no formal review with partners.^31^

These mechanisms were selected because they are frequently cited as examples of independent oversight in global health.^22,32,33^ The table below (Table 1) outlines the OPEN framework, and below we summarize our assessment of each initiative against it. For a more detailed explanation of the scoring of each across the 4 dimensions of independence, see Appendix Table S1.

**Table 1.** OPEN Framework.

Dimension	Criteria	Rating (1 = Yes, 3 = Partially/not sure, 5 = No)
Operational/Organizational	Full freedom over the hiring process for staff	1: The committee has full authority to define roles, recruit, and appoint its secretariat or contractors. 3: The committee participates in hiring decisions, but appointments require approval from the host or other external authorities. 5: Appointments are managed externally, with little to no decision-making power retained by the committee.
	Set own work program and scope to produce reports and analysis	1: The committee determines its own priorities, scope of work, and schedule for analysis and reporting. 3: The committee can propose its work program, but final approval or revisions are made by the host or other external actors. 5: The committee's work program, scope, and schedule are set externally.
	Ability to disclose without management-imposed restrictions or those whose activities it evaluates	1: The committee can publish findings, reports, and statements without prior clearance or limitations by the host or other external actors. 3: There are informal expectations or diplomatic pressures that may shape disclosure. 5: The committee must obtain approval before publication or statement.
	Operates autonomously and is not subject to organizational policies or pressures of its hosting/supporting organization	1: The committee operates at arm's length from the hosting organization and is not subject to directives, organizational policies, or hierarchical pressures from its host. 3. The hosting organization has influence over the committee operations (eg, funding, policies, procedures, tenders) 5. Operational decisions must be approved by the hosting organization.
Political	Defined criteria for appointment and dismissal	1: The process for appointing and dismissing committee members is governed by clear, publicly available criteria. 3: Appointment and dismissal procedures exist, but they are not publicly available. 5: Criteria for appointing or dismissing committee members do not exist.
	Diplomatic immunity to protect committee members from arrest or prosecution for acts taken in carrying out official duties.	1: Committee members are granted formal legal protections, such as diplomatic immunity or other legal safeguards. 3: Legal protections may exist in principle, but are not actively applied. 5: Committee members lack any form of legal immunity or protection.
Economic/Financial	Does not have a material relationship with the host institution or have a material relationship with a related company that conducts business with the company	1: The committee has control over its budget and expenditures. 3: The committee has some financial autonomy, but its operations are subject to funding from the host. 5: The committee does not control its budget or expenditures.
	Firewalled from engagement with any donors or group of donors	1: The committee and its members are prohibited from receiving funding from the organizations they assess. 3: The committee does not routinely receive funding from the organizations they assess, but there are no formal prohibitions in place. 5: The committee or its members receive funding, directly or indirectly, from organizations they are tasked with assessing,
	Funded through assessed or non-earmarked funding and, therefore, not subject to pre-conditions through which countries and other donors can impose their individual priorities or political considerations	1: The committee is fully funded through assessed or non-earmarked contributions. 3: The committee is partially funded through assessed or non-earmarked contributions. 5: The committee relies on earmarked or conditional funding tied to donor-defined objectives.
Knowledge/ Technical	Objective scientific assessment of a state party free from undue influence from financial and political actors (mainly state parties and donors) meant to distort or bias the conduct or findings of an evaluation	1: The committee is composed of experts serving in a personal capacity, with no institutional dependencies to compromise objectivity. 3: There is no direct evidence of influence; however, the committee's proximity to financial and political actors may create dependencies. 5: The committee's experts do not serve in individual capacity, or institutional dependencies create a conflict of interest.
	Access to information and expertise in public health	1: The committee can freely collect complementary information and consult external sources, including the UN, academia, and CSOs, to support or verify information. 3: The committee has access to some information and expertise, with limited ability to collect or validate data from external sources. 5: The committee relies entirely on information provided by a single institution (eg, host agency, state).
	Necessary configuration and level of power to conduct periodic and ad hoc inquiries and visits	1: The committee can conduct planned and unannounced on-site visits to verify information. It has the authority to seek information from relevant parties through direct inquiries. 3: The committee can conduct planned visits at the invitation of the state and seek information from relevant parties through direct inquiries. 5: The committee does not conduct on-site visits, nor does it have the authority to conduct direct inquiries.

The IMB enjoys a high level of independence both in its structure and its operations. This is due, in part, to the fact that IMB findings are non-public. The committee's findings are submitted to the WHO Director-General and the Executive Board. Therefore, IMB findings are filtered through other bodies before being made public. This encourages frank disclosure but gives them less direct influence on state behavior and therefore makes them less likely to have their independence targeted and curtailed. The lack of remuneration for IMB members could contribute to a narrowing of candidates able to accept an appointment, excluding candidates without personal resources to work for free and/or those from low-income countries where salaries may not support taking the posting. Funding is from a pooled source and is not dependent on any one donor.

The GPMB’s functional independence is limited due to its interwoven relationship with WHO and the World Bank, which provide its funding and appoint its members. The GPMB's work is commissioned by the WHO Secretariat, and its findings are communicated via the WHO. GPMB members include individuals from donor organizations, such as the Gates Foundation. Committee members are subject to WHO policies, including a 60-day notice period requirement for members publishing elsewhere on their oversight work. The GPMB's independence would be substantially enhanced by several structural changes: allowing it to publish directly the results of its findings, updating how its members are appointed, and providing alternative funding arrangements. It would also benefit from being structurally independent from the WHO, as WHO is integral to many of the activities being assessed.

The IAP had significant independence in producing its work product; however, its staffing and funding arrangements were not optimal for independence. Members served in a pro bono capacity, which introduced potential conflicts, as they were supported by their home institutions. Some members of the IAP held positions in government, further complicating perceptions of impartiality. Furthermore, the committee was staffed and funded by its host, PMNCH, which in turn is hosted by WHO. While independent, its reliance on WHO systems as a fiduciary, legal, and administrative agent limits full operational autonomy. Given the wide dissemination of IAP reports and findings, this could have inhibited members' ability to provide unfiltered contributions.

## Conclusion

Given the limited set of actors, conflict and confluence of interests are at work in the execution of most programs in public health. It can be difficult to find experts with no stake in the game. However, it is possible to set guiderails and guardrails that can standardize practice and contribute to independent evaluation and monitoring, which are critical to credibility, confidence, transparency, accountability, performance assessment, and learning.

Recognizing that it may not be possible to incorporate all aspects of independence in an oversight committee, we propose using the OPEN framework as the industry standard in global public health. While this framework was developed to understand the practice of independent monitoring, it could also be applied more broadly to any function that requires independence, including auditing and evaluation.

While independent oversight and monitoring committees should aim to score highly against all criteria, in cases where this is not possible, OPEN provides a systematic approach to identify potential conflicts and develop mitigation strategies, where the criteria cannot be met. While some criteria are non-negotiable, such as the freedom to conduct direct inquiry or funding not tied to a particular donor or group of donors, others may be possible to mitigate. For example, if staffing is provided by the host organization, which may have a conflict in that it also indirectly or directly involves the subject of evaluation, the staff could develop dual reporting lines both to the host organization and the committee. Or if a donor or former member of government from a particular country is part of the committee, that member can be recused from decisions related to the work of their organization or former country.

While absolute independence will never be possible given the complex interdependencies and relationships among those engaged in global health, applying the OPEN framework can improve oversight committees to achieve greater objectivity, impartiality, and impact by forcing an honest conversation about conflicts of interest and a systematic assessment of actions to mitigate those conflicts. Using the OPEN framework across these 4 dimensions (organizational/operational, political, economic/financial, and knowledge/technical) can improve transparency, foster trust, and ultimately improve public health outcomes by enhancing the credibility of assessment on performance, progress, and investments that honestly assess external pressure, bias, and conflict of interest.

## Supplementary Material

qxaf231_Supplementary_Data
