# Supplementary material for: Securing independence in global health oversight—the OPEN framework
Source: Health Aff Sch. 2025 Nov 27;3(12):qxaf231. doi: 10.1093/haschl/qxaf231 (PMC12684966; doi:10.1093/haschl/qxaf231)
Supplement: qxaf231_Supplementary_Data [file qxaf231_supplementary_data.zip › Supplemental Material Appendix I fin.docx]

**Appendix I – Table A1: Independence Assessment Scores for Reviewed Entities**^1^

| **Criteria** | **IMB**^2–4^ | | **GPMB**^5,6^ | | **IAP**^7,8^ | |
| --- | --- | --- | --- | --- | --- | --- |
|  | **Score** | **Comments** | **Score** | **Comments** | **Score** | **Comments** |
| **Operational/ Organizational** | | | | | | |
| Full freedom over the hiring process for staff | 1 | The chair has a local secretariat, while the WHO may provide additional support. The chair leads the appointments of the team (currently 4 members), the WHO Director General (DG) confirms, the Board is consulted. | 5 | The Secretariat is staffed by WHO. WHO and the World Bank appoint co-chairs outside their organizations as well as members. | 5 | The Secretariat is staffed by PMNCH. Members, including the co-chairs, are appointed by the UN Secretary-General. |
| Set own work program and scope to produce reports and analysis | 3 | Scope yes, but not the program (e.g., schedule) as that is set from the outset (meets twice a year for 3 days). | 3 | The annual key report is defined from the outset, but can produce additional communications. | 1 | Yes. Also, regularly assesses its impact, drawing lessons learned and introducing improvements. Members will periodically conduct a self-evaluation of its functions, as individual members, and a collective evaluation of the panel as a whole. |
| Ability to disclose without management-imposed restrictions | 1 | WHO, local secretariat, other partners, or states do not interfere. Draft reports are not shared with countries, so no mechanism to influence, IMB writes reports itself. Reports feed into WHO DG and EB reports, but IMB doesn’t have to think about that when reporting. | 5 | No. The Secretariat (WHO) issues any public communication, including through the WHO website. WHO owns copyright. Members that publish anything separately shall share with WHO and the World Bank 60 days before all materials intended to be published. | 1 | Disseminates its recommendations and reports widely to Member States and other stakeholders. |
| Operates autonomously and is not subject to organizational policies or pressures of its hosting/supporting organisation | 1 | Yes, as not “hosted” as such, works independently and is not tied to WHO policies. | 5 | Subject to WHO policies. Convened by WHO and the World Bank, both organizations are subject to their own evaluation. | 3 | PMNCH/WHO policies do not interfere with the execution of duties. However, members operate on a pro bono basis, with some members possibly from government. |
| **Political** | | | | | | |
| Defined criteria for appointment and dismissal | 1 | Yes, outline in the ToR (criteria and probity). | 3 | To some extent. Expertise listed. WHO Conflict of Interest applied. No dismissal criteria. | 3 | To some extent. Criteria for appointment, but not for dismissal. |
| Diplomatic immunity to protect committee members from arrest or prosecution for acts taken in carrying out official duties | 5 | No. | 5 | No. “WHO shall not assume any liability for acts carried out by Board members.” | 5 | No. |
| **Economic/Financial** | | | | | | |
| Does not have a material relationship with the host institution or have a material relationship with a related company that conducts business with the company | 1 | Does not have a host as such. | 5 | Subject to the availability of sufficient human and financial resources provided by WHO. | 5 | PMNCH, as a host, approves the budget and serves as the primary source of financing for the IAP. |
| Firewalled from engagement with any donors or group of donors | 1 | Members cannot be from GPEI partner organizations. | 5 | Donors (e.g., BMGF) are members. | 1 | Members cannot be employees of the United Nations and its specialized agencies. |
| Funded through assessed or non-earmarked funding and, therefore, not subject to pre-conditions through which countries and other donors can impose their individual priorities or political considerations | 1 | Through GPEI. Members do not receive remuneration; only travel expenses are compensated. | 5 | Not clear. Staff on WHO contracts and funded in part by WHO. | 1 | While PMNCH approves the budget and serves as the primary source of financing for the IAP, *activities* related to this budget will be solely at the discretion of the IAP Secretariat and not require PMNCH’s approval. Members do not receive remuneration. |
| **Knowledge/Technical** | | | | | | |
| Objective scientific assessment of a state party free from undue influence from financial and political actors (mainly state parties and donors) meant to distort or bias the conduct or findings of an evaluation | 1 | Members serve as individuals, independent from GPEI partners and states. | 3 | Consists of high-level (e.g. agency principals, CEOs, etc.) members from across relevant stakeholders with public and/or private sector experience. | 1 | Consists of independent experts who do not seek or accept instructions from any other party. |
| Access to information and expertise in public health | 1 | Yes. | 1 | Yes. | 1 | Yes. Utilizes a range of sources, including the UN, academia, CSOs, and independent bodies. |
| Necessary configuration and level of power to conduct periodic and ad hoc inquiries and visits | 3 | Can conduct inquiries. Planned visits possible (not necessary) with a country’s permission, no unannounced visits. | 5 | Works through WHO. Travel not mentioned and if it would have, is limited by WHO policies and procedures (i.e., by state invite only). | 3 | Invites stakeholder contributions and evidence that may inform reviews. Travel not mentioned. In practice, does not have the power to conduct unannounced visits. |

## References

1. Spark Street Advisors, UNU-IIGH. *Independent Monitoring for the Pandemic Accord*. UNU-IIGH; 2023. https://collections.unu.edu/eserv/UNU:9304/Independent-Monitoring-for-the-Pandemic-Accord.pdf

2. Global Polio Eradication Initiative. Independent Monitoring Board. 2025. Accessed May 14, 2025. https://polioeradication.org/who-we-are/governance/independent-monitoring-board/

3. Global Polio Eradication Initiative. *Global Polio Eradication Initiative Independent Monitoring Board and Transition Independent Monitoring Board*.; 2020. Accessed May 14, 2025. https://www.archive.polioeradication.org/wp-content/uploads/2020/02/ToR-IMB-TIMB-Jan2020-Dec2021-20200122.pdf

4. Rutter PD, Donaldson LJ. Oversight Role of the Independent Monitoring Board of the Global Polio Eradication Initiative. *J Infect Dis*. 2014;210(suppl 1):S16-S22. doi:10.1093/infdis/jiu181

5. GPMB. *Global Preparedness Monitoring Board*. WHO; 2025. Accessed May 14, 2025. https://www.gpmb.org/

6. GPMB. *Terms of Reference Global Preparedness Monitoring Board*.; 2021. Accessed May 14, 2025. https://www.gpmb.org/docs/librariesprovider17/default-document-library/gpmb-tors.pdf?sfvrsn=36edfabe_3

7. Independent Accountability Panel. IAP at a glance. 2021. Accessed September 7, 2021. https://iapewec.org/about/iapataglance/

8. Independent Accountability Panel. Terms of Reference. 2018. Accessed August 17, 2023. https://iapewec.org/wp-content/uploads/2018/12/IAP-TORs_updated_Sept2018-2.pdf
